# Development of Solid Lipid Nanoparticles for Controlled Amiodarone Delivery

**DOI:** 10.3390/mps6050097

**Published:** 2023-10-09

**Authors:** Andreea Creteanu, Gabriela Lisa, Cornelia Vasile, Maria-Cristina Popescu, Adrian Florin Spac, Gladiola Tantaru

**Affiliations:** 1 Department of Pharmaceutical Technology, Faculty of Pharmacy, “Grigore T. Popa” University of Medicine and Pharmacy, 16 Universitatii Street, 700115 Iași, Romania; acreteanu@gmail.com; 2 Department of Chemical Engineering, Faculty of Chemical Engineering and Environmental Protection, “Gheorghe Asachi” Technical University, 73 Prof. Dr. Docent Dimitrie Mangeron Street, 700050 Iași, Romania; gapreot@yahoo.com; 3 Physical Chemistry of Polymers Department, Petru Poni Institute of Macromolecular Chemistry, 41A Gr. Ghica Voda Alley, 700487 Iași, Romania; cvasile@icmpp.ro (C.V.); cpopescu@icmpp.ro (M.-C.P.); 4 Department of Phisico Chemistry, Faculty of Pharmacy, “Grigore T. Popa” University of Medicine and Pharmacy, 16 Universitatii Street, 700115 Iași, Romania; 5 Department of Analytical Chemistry, Faculty of Pharmacy, “Grigore T. Popa” University of Medicine and Pharmacy, 16 Universitatii Street, 700115 Iași, Romania; gtantaru2@yahoo.com

**Keywords:** amiodarone, solid lipid nanoparticles, controlled delivery

## Abstract

In various drug delivery systems, solid lipid nanoparticles are dominantly lipid-based nanocarriers. Amiodarone hydrochloride is an antiarrhythmic agent used to treat severe rhythm disturbances. It has variable and hard-to-predict absorption in the gastrointestinal tract because of its low solubility and high permeability. The aims of this study were to improve its solubility by encapsulating amiodarone into solid lipid nanoparticles using two excipients—Compritol^®^ 888 ATO (pellets) (C888) as a lipid matrix and Transcutol^®^ (T) as a surfactant. Six types of amiodarone-loaded solid lipid nanoparticles (AMD-SLNs) were obtained using a hot homogenization technique followed by ultrasonication with varying sonication parameters. AMD-SLNs were characterized by their size distribution, polydispersity index, zeta potential, entrapment efficiency, and drug loading. Based on the initial evaluation of the entrapment efficiency, only three solid lipid nanoparticle formulations (P1, P3, and P5) were further tested. They were evaluated through scanning electron microscopy, Fourier-transform infrared spectrometry, near-infrared spectrometry, thermogravimetry, differential scanning calorimetry, and in vitro dissolution tests. The P5 formulation showed optimum pharmaco-technical properties, and it had the greatest potential to be used in oral pharmaceutical products for the controlled delivery of amiodarone.

## 1. Introduction

In the treatment of chronic human diseases, taking into account the site-specific and target-oriented delivery of precise medicines, many benefits are offered by nanotechnology. Compared to other colloidal carriers, solid lipid nanoparticles (SLNs) offer many benefits, among which, we can mention the fact that they can be formulated as self-administrable eye drops, are sterilizable, and are biocompatible; their production steps are simple and easy to expand on a large scale; they have a high entrapment efficiency and show long-term stability; and, last but not least, they show controlled drug release and site-specific drug delivery [1]. In addition, in the case of sensitive lipophilic drugs, they can be protected against degradation by the fact that they are immobilized by SLNs in the solid state of the lipid matrix [2].

Used as nanocarriers, SLNs present a remarkable potential for the administration of drugs via different routes (such as oral, nasal, ocular, pulmonary, rectal, vaginal, transdermal, or parenteral) [3,4,5]. Taking into account their nanometric size [6] and the special properties they present (such as surface charge, mass, density [7,8], etc.), an advantage of SLNs is the formation of a relatively large surface, a fact that facilitates the binding of various ligands, including antibodies, aptamers, peptides, DNA, RNA [9,10,11,12,13], and others, which helps release the modified SLNs at the site of action (in vivo).

Amiodarone hydrochloride (AMD) is an antiarrhythmic agent used in severe rhythm disturbances, such as symptomatic confirmed cases of ventricular rhythm disturbances and less severe ventricular arrhythmias, including atrial fibrillation, tachycardia in Wolff–Parkinson–White syndrome, and supraventricular rhythm disturbances with a rapid ventricular rhythm [14,15].

Within the Vaughan Williams classification, AMD belongs to the Class III antiarrhythmic drugs, but it possesses multiple electrophysiological effects (which is characteristic of all four Vaughan Williams classes). Although it generates thyroid dysfunction and liver or lung toxicity [16,17,18], AMD remains one of the most effective drugs for the treatment of a wide range of arrhythmias because it has a high clinical efficacy, including in patients who are refractory to the administration of adequate doses of other antiarrhythmic drugs or have complications due to Wolff–Parkinson–White syndrome [15,19].

AMD is one of the most frequently prescribed therapeutic agents for cardiac tachyarrhythmias in the world due to its recognized effectiveness [20,21]. It remains the most effective drug for prophylaxis and treatment of many types of arrhythmias, as it is sometimes the only available option in the treatment of arrhythmias [19].

AMD shows a variable and thus hard-to-predict absorption in the gastrointestinal tract. This unpredictable absorption can be explained by the hepatic first-pass effect and by the reduced solubility of AMD in aqueous solutions (0.2–0.7 mg/mL) [22]. Currently, in AMD cardiovascular therapy, it is used in the form of an injectable solution or tablets with conventional release (200 mg/tablet), with the recommendation that it be administered in 2–3 doses/day [15]. Orally administered AMD is slowly and partially absorbed. The relatively limited bioavailability with large individual variations is caused by the dealkylation of the molecule in the intestinal mucosa [23]. The second class of the biopharmaceutical classification system (BCS) contains substances with low solubility, high permeability, and a relatively low oral bioavailability; therefore, their solubility must be improved. For example, to improve the substances’ solubility and drug release, their inclusion in complexes with cyclodextrin or its derivative 2-hydroxypropyl-β-cyclodextrin [24,25] or encapsulation in solid lipid nanoparticles (SLNs), as in the case of trans retinoic acid [26], can substantially optimize oral therapies using drugs with low solubility.

In research on the oral administration of drugs, great attention is paid to lipid nanocarriers, which include nanostructured lipid carriers and SLNs. In the case of SLNs, they are composed of various biodegradable lipids, such as highly purified monoglycerides or triglycerides or complex mixtures of glycerides, solid fats, and some waxes that are in a solid state at physiological temperatures. The ability to support therapeutic levels of AMD is due to the presence of the solid lipid phase of SLNs [27,28,29].

Regarding the improvement of drug permeability in biological tissues to lead to an increase in overall efficacy, promising results were obtained in studies using lipid-based nanoparticle systems [30,31]. SLNs are carriers of nanometric lipids, with dimensions in the range of 50–1000 nm, which have a capacity to encapsulate different drug molecules inside their lipid matrix [32]. Thus, the drug molecule is distributed in the crystalline lipid lattice, which leads to an increase in the general stability of the formulation. Another advantage of SLNs is their ability to increase the solubility of hardly soluble or insoluble molecules and/or to increase the permeability of molecules with low permeability [30]. In general, conventional SLNs containing BCS Class II drugs are obtained using surfactants acting as penetration/permeation enhancers and solubilizers. Their addition during the preparation of lipid nanoparticles contributes to the lowering of the melting enthalpy of Compritol 888 ATO (C888) by distributing the molten lipid phase and distorting its crystallization [33]. The in vitro data have indicated that, although commonly employed surfactants and endogenous surfactants present in the intestines can enhance drug solubility, most of them decrease drug permeation [29].

Therefore, the solid lipid nanoparticles (SLNs) are considered particularly useful nanocarriers for the administration of lipophilic drugs. Many different drugs have been incorporated into SLNs, such as timolol, doxorubicin diazepam, progesterone, hydrocortisone, paclitaxel, vitamin E palmitate, aciclovir, tetracaine, cyclosporin, nimesulide, oxazepam, etc. [32]. They present many advantages, including suitability for large-scale production, high stability with minimal drug leakage, minimal physiological toxicity, high drug-loading capacity, drug targeting, and controlled and/or sustained drug release. The lipophilic nature and small size of the particles makes them effective in penetrating physiological barriers. However, so far, there are no studies to obtain amiodarone-loaded SLNs.

The aim of the present study was to obtain a new modified receipt for AMD-loaded SLNs (AMD-SLNs) of a select composition, including Compritol^®^ 888 (C888) (pellets) as the lipid matrix and Transcutol^®^P (T) surfactant as the penetration enhancer of the AMD, through the hot emulsification–ultrasonication technique followed by its detailed physical–chemical and pharmaco-technical characterization.

Compritol^®^ 888 ATO is based on glycerol esters of behenic acid (C22): 12–18% glycerol monobehenate, 52–54% glycerol dibehenate, and 28–32% glycerol tribehenate. Behenic acid is its main fatty acid (>85%), but other fatty acids (C16–C20) are also present. In the preparation of SLNs for the capture of lipophilic drugs, one of the most applied and cited excipients is C888. The choice of this lipid excipient took into account the fact that its incorporation with other constituents in the receipt could ensure the formation of SLNs with a high drug-loading capacity, with small dimensions and high stability. A possible explanation of its effect could be the presence of some chains of variable length in its structure, which form a disordered crystalline lattice in whose net the drug substance is fixed [34,35].

Diethylene glycol monoethylether (Transcutol^®^), a well-known and effective permeation enhancer, is soluble in both water and oil, nontoxic, compatible with biological tissues, and used in several pharmaceutical formulations. Transcutol^®^P, playing the role of both surfactant and solvent, can increase the solubility of some drugs that are slightly soluble in water.

This excipient was chosen because it led to the formation of stable AMD-SLNs, with small dimensions, but also because it ensures a high drug-loading capacity, as we mentioned above, due to the presence of chains with variable lengths in the structure, which form a disordered crystalline network in which the drug is encapsulated [35]. Select concentrations of the lipid ingredients, T 2% (*w*/*w*) and C888 5% (*w*/*w*), were established according to the literature data for other kinds of drugs [34,35,36].

Various factors, such as the components that are part of the formulation, their solubility (including the drug), and the production method, influence the structure of SLNs. The solid state of the particle (e.g., crystallization and physicochemical transitions) influences the modified release property. Retarded or suppressed crystallization has been reported, in which cases a poor drug was obtained. The loading capacity is due to the high hydrophobic character of the charged molecules but also due to the imperfect crystalline structure produced by Compritol^®^ during recrystallization, which reduces the exclusion of the drug from the lipid matrix [37]. In conclusion, the method of preparation, the composition of the lipid matrix, and the surfactant used determined the characteristics of the solid lipid nanoparticles obtained.

The ratio between AMD, the lipid excipient C888, and T was maintained constant, but various ultrasonication conditions were applied. Additionally, empty control SLNs (without AMD) were prepared using the same procedures and the same surfactant concentration as the loaded formulations.

To evaluate the effect of the parameters of the sonication process on the AMD-SLNs’ characteristics, the preparation was conducted by varying the sonication cycles and amplitude. The obtained AMD-SLNs were characterized in terms of morphology, mean particle size, polydispersity index (PDI), zeta potential (ZP), entrapment efficiency (EE%), and drug loading (DL%) through scanning electron microscopy (SEM), Fourier-transform infrared spectrometry (FT-IR), near-infrared spectrometry (NIR), thermogravimetry (TG), differential scanning calorimetry (DSC), and in vitro dissolution tests, in order to assess the most suitable preparation conditions.

## 2. Materials and Methods

### 2.1. Materials and Instruments

Amiodarone hydrochloride of 99.85% purity was purchased from Zhejlang Sanmen Hengkang Pharmaceutical Co., Ltd. (Hubei, China). Compritol^®^ 888 ATO (C888) and Transcutol^®^ (T) were purchased from Gattefossé (Lyon, France). All other reagents and solvents of analytical grade were purchased from Merck (Darmstadt, Germany). The used instruments were a high-shear homogenizer (Ultra Turrax T25 IKA, Wilmington, NC, USA), ultrasound bath Soniprep 150 (MSE Crowley, London, UK), centrifuge (AccuSpin 17R, Fisher Scientific, Hanover, IL, USA), centrifugal filter unit (Amicon Ultra, Fisher Scientific, Hanover, IL, USA), electronic thermostat Evco (EV3401P7, Sedico BL, Italy), scanning electron microscope type Verios G4 UC (Thermo Scientific, Brno, Czech Republic), HPLC with diode-array detector (both from Thermo Fisher, San Jose, CA, USA), chromatographic column (Thermo Fisher–Hypersil Betasil) C18 column, 150 mm × 4.6 mm, 5 µm particle size (Thermo Fisher, San Jose, CA, USA), dynamic light-scattering Nano ZS Zetasizer apparatus (MicrotracBEL, Malvern Instruments, Enigma Business Park, Malvern, UK), Bruker Alpha Platinum ATR spectrometer (Bruker, Ettlingen, Germany), PHAZIR handheld near-infrared analyzer (Thermo Fisher Scientific—Portable Optical Analysis, Waltham, MA, USA) with Grams 9.1 software (Thermo Fisher Scientific-Waltham, MA, USA), thermogravimeter Mettler Toledo 851e (Columbus, OH, USA) with STAR software 16.30.13258 (Mettler Toledo, Columbus, OH, USA), and dissolution test station type II, Hanson SR 8 Plus Series (Hanson Research Co., Chatsworth, CA, USA) with baskets.

### 2.2. Preparation of AMD-SLNs

AMD-SLNs were prepared via hot homogenization followed by ultrasonication using the method described by Bose et al. [38], with some modifications.

The concentrations of the different components in SLN formulations were chosen on the basis of a preliminary screening (preformulation study) that had been set up to find the optimized concentration for each component [39]. The different components that assured the higher encapsulation efficiency of SLN were C888 5% (*w*/*w*), T 2% (*w*/*w*), and AMD (as the encapsulated compound) 0.13% (*w*/*w*). SLNs were obtained by melting C888 at 70 °C and creating a hot aqueous solution containing AMD, then adding T 2% (*w*/*w*) of the final SLN dispersion in 50% (*w*/*w*) water. The pre-emulsion was homogenized using a high-shear homogenizer for 5 min at 650 rpm. Cold water (rest until 100 g mixture, *w*/*w*) was added to the pre-emulsion and then sonicated for 4 min (0.5–1 cycles, 5 s “on”, 2 s “off”) at 40, 70, and 100% amplitude, thermostated at 85 °C. The obtained hot AMD/W nanoemulsion was cooled down to room temperature, and the sonication of the mixture continued at room temperature for 10 min. In the final mixture, solid particles appeared, forming a suspension.

The ratio between AMD, the lipid excipient C888, and T was maintained constant, but various ultrasonication conditions were applied (the amplitude was varied from 40 to 100% and the ultrasonication was varied from 0.5 to 1 s). Additionally, empty control SLNs were prepared using the same procedure and the same surfactant concentration as loaded formulations. The obtained samples were stored at 25 °C prior to use and characterized 24 h after preparation.

To evaluate the effect of sonication on PDI and size of AMD-SLNs, six types of AMD-SLNs were obtained, P1, P2, P3, P4, P5, and P6, varying amplitude and sonication cycles, as shown in Table 1.

### 2.3. Scanning Electron Microscopy (SEM) Analysis

The form and morphological characteristics of the AMD-SLNs were studied using a scanning electron microscope type Verios G4 UC working in STEM mode at 20 kV, with a STEM 3+ detector (bright-field mode) and without any further treatments, at 20.000 magnification (indicated in figures). One drop of each suspension was placed on carbon-coated copper grids with a 300 mesh size and allowed to dry at room temperature (*c.a.* 23 °C).

### 2.4. Drug-Loading Capacity (DL%) and Entrapment Efficiency (EE%) of the AMD into AMD-SLNs

Quantitative determination of the drug-loading capacity (*DL*%) of the SLNs with AMD was performed using the lipid precipitation method. First, 0.5 mL of the AMD-SLN suspension was precipitated with 4.5 mL of methanol, followed by centrifugation at 600 rpm for 10 min. Then, 1.0 mL of the supernatant was diluted to 5 mL with mobile phase (a mixture of 0.5% formic acid in phosphate-buffered solution pH = 7.6/methanol (25/75, *v*/*v*)), resulting a 1.3 mg/mL AMD solution that was analyzed using a validated HPLC method with UV detection at 254 nm [20].

*DL*% in the AMD-SLNs was calculated using the formula

(1) $$DL\%=\frac{{AMD}_{t}-{AMD}_{u}}{AMD-SLNs}\times 100$$

where *AMD_t_* is the total weight of the drug, *AMD_u_* is the weight of the unloaded drug, and *AMD-SLNs* is the weight of the *AMD* − *SLNs*.

The EE% in the AMD-SLNs was calculated by estimating the concentration of the free drug in the aqueous phase of an undiluted formulation, using an ultrafiltration method with a 100 kDa centrifugal filter. A 500 μL aliquot of the formulation was added to the filter unit and centrifuged at 600 rpm for 10 to 15 min. Then, 1.0 mL filtrate was diluted to 5 mL with mobile phase and analyzed using HPLC with UV detection. The EE% was calculated using the formula

(2) $$EE\%=\frac{{AMD}_{t}-{AMD}_{f}}{{AMD}_{t}}\times 100$$

where *AMD_t_* is the total drug content and *AMD_f_* is the free drug content present in the aqueous phase.

HPLC analysis was performed using UV-Vis by means of a Thermo Fisher Surveyor chromatograph equipped with a multiple-diode-array detector and a Thermo Fisher–Hypersil Betasil C18 column, 150 mm × 4.6 mm, 5 µm particle size. The column temperature was kept constant at 45 ± 0.2 °C. A mobile phase was used, a mixture of 0.5% formic acid in phosphate-buffered solution (pH = 7.6)/methanol (25/75, *v*/*v*) at a flow rate of 0.7 mL/min. The injection volume for each determination was 20 μL. AMD detection was performed at the wavelength of 254 nm. The recorded retention time for AMD was 4.51 min [32].

### 2.5. Dynamic Light Scattering (DLS) for Polydispersity Index (PDI) and Zeta Potential (ZP) Measurements

The particle size, PDI, and ZP of the AMD-SLNs were analyzed through the DLS method using a Nano ZS Zetasizer apparatus equipped with a He-Ne laser (λ = 633 nm) and operated at a scattering angle of 173°. AMD-SLNs were analyzed after 1/20 dilution with water.

### 2.6. FT-IR Spectroscopy Analysis

The FT-IR spectra of the pure AMD, lipid excipient, surfactant, and AMD-SLNs from the suspension were recorded in the 500 to 4000 cm^−1^ wavenumber range with a resolution of 2 cm^−1^, with 64 scans, by means of a Bruker Alpha Platinum ATR spectrometer in absorbance mode.

### 2.7. Near-Infrared (NIR) Spectroscopy Analysis

NIR spectra were recorded by means of a PHAZIR handheld near-infrared analyzer in the spectral range of 900–2600 nm using the diffuse reflectance method. In order to evaluate the reproducibility of the data, for each AMD-SLN, five spectra were recorded. Processing of the spectra was performed using the Grams 9.1.

### 2.8. Thermogravimetry

The thermal behavior analysis for AMD-SLNs and AMD, C888, and T was performed using Mettler Toledo 851e equipment in a nitrogen atmosphere, with a rate of 10 °C/min. The thermogravimetric (TG) and derivative thermogravimetric (DTG) curves were recorded in the temperature range of 25–700 °C. The processing of the TG and DTG curves for obtaining the main characteristics was performed using STAR software.

### 2.9. Differential Scanning Calorimetry (DSC) Analysis

The DSC curves were recorded with a Mettler Toledo DSC1 apparatus in an inert atmosphere (nitrogen) with a 10 °C/min heating rate. Scans were performed in the temperature range of 25–160 °C for the AMD-SLNs (P1, P3, and P5), 25–200 °C for AMD, and 25–230 °C for C888. In order to highlight the presence of AMD in the structures of the AMD-SLNs, scans were also performed in the temperature range of 25–200 °C with a heating rate of 10 °C/min. The masses of the AMD-SLNs subjected to analysis were between 2 and 11 mg.

### 2.10. In Vitro Dissolution Tests of AMD from AMD-SLNs

The AMD dissolution profiles were studied using two dissolution media: 0.1 N HCl solution with pH = 1.2 as the simulating medium for gastric fluids (SMGF) and phosphate-buffered solution with pH = 6.8 as the simulating medium for intestinal fluids (SMIF). The experiments were carried out by means of a dissolution test station type II, Hanson SR 8 Plus Series with baskets. The experiments were performed according to the requirements described in the Romanian, European, and United States Pharmacopoeias [40,41,42]. The dissolution test was performed at 37 ± 0.5 °C and 60 rpm, with 500 mL dissolution medium SMGF for the first two hours of the test and then SMIF for the next 34 h. The mass of the AMD-SLNs was equivalent to 100 mg AMD. Aliquots (2 mL) of the dissolution medium were collected for HPLC analysis and replaced with fresh dissolution medium every 30 min for 36 h. The AMD was quantitatively determined using the validated HPLC method previously described [43]. Tests were performed in triplicate and the average value was calculated.

### 2.11. Kinetic Release of AMD from AMD-SLNs

The kinetic release parameters were calculated using the Korsmeyer–Peppas equation [44]:

(3) $$\frac{M_{t}}{M_{\infty }}=k_{r}\times t^{n_{r}}$$

where *M_t_*/*M_∞_* represents the fraction of the drug released at time *t*, *k_r_* is a constant incorporating the characteristics of the macromolecular matrix, and *n_r_* is the diffusion exponent, which is indicative of the release mechanism. In the equation above, a value of 0.5 or smaller for *n_r_* indicates a Fickian diffusion mechanism of the drug from the matrix. A value of *n_r_* between 0.5 and 1 indicates an anomalous or non-Fickian behavior. When *n* = 1, a case II transport mechanism is involved. Values *n* > 1 indicate a special case II transport mechanism.

## 3. Results and Discussions

### 3.1. Preparation of AMD-SLNs

Six AMD-SLN samples (P1, P2, P3, P4, P5, and P6) were prepared via homogenization and ultrasonication. The ratio between AMD and the lipid excipient C888 remained constant, but various ultrasonication conditions were applied (amplitude and ultrasonic cycles, as shown in Table 1).

### 3.2. Scanning Electron Microscopy (SEM) Results

**Figure 1:**
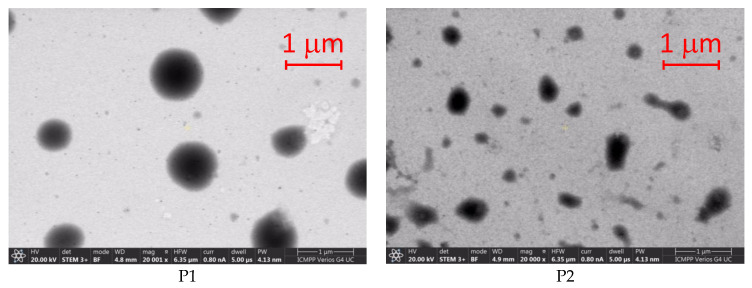

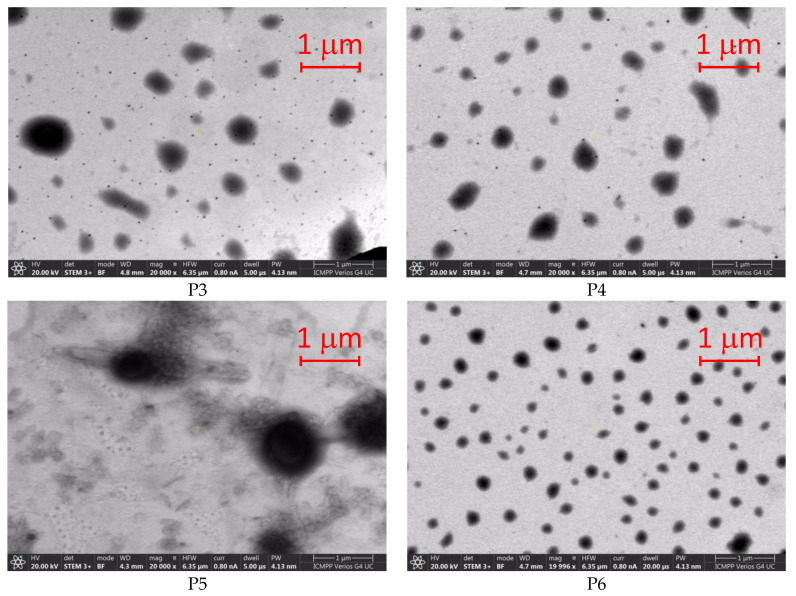
Scanning electron microscopy (SEM) microphotographs of amiodarone hydrochloride-loaded solid lipid nanoparticles (AMD-SLNs) resulting from the variation in the ultrasonication parameters. Magnification 20,000×.

The scanning electron microphotographs of the physical mixture of the AMD-SLN spheres are shown in Figure 1. The images reveal that the freshly prepared nanoparticles were almost spherical in shape, with a smooth surface, without visible aggregations between them. In the physical mixture, no interactions took place between the AMD, the lipid matrix, and the surfactants. The morphology of the AMD-SLNs appeared to be irregular sheets with a large surface area. The SLN micrographs also suggest the presence of an amorphous homogeneous phase.

P5 and P6 formulations were spherical, smooth, uniform particles with no imperfections and no cracks.

**Figure 2:**
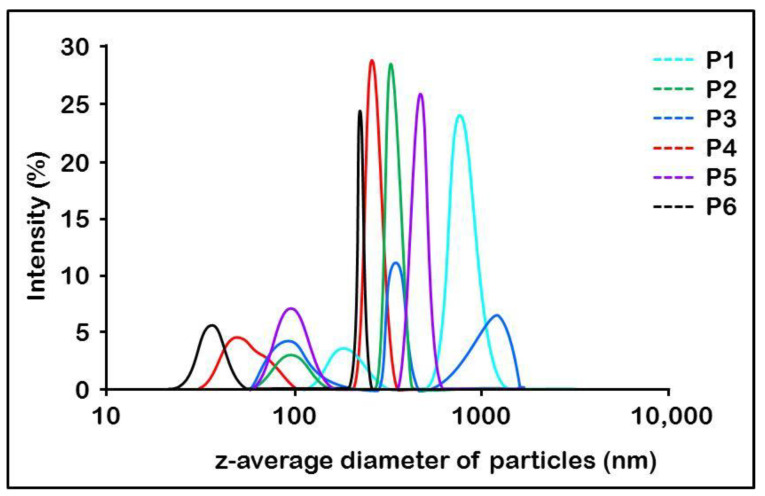
Particle size distribution of AMD-SLNs.

The particle size and the distribution are specific for each sample, as at least two or three different sizes are clearly present in the distribution curves obtained through the dynamic light-scattering investigation method, as seen in Figure 2.

### 3.3. Drug-Loading Capacity (DL%) and Entrapment Efficiency (EE%) of the AMD in AMD-SLNs

An important factor to be considered in the optimization of AMD-SLNs was the amount of drug that was encapsulated in the nanoparticles and the drug content in the lipid matrix. Upon heating, the drug dissolved in the molten lipid matrix, and the amount of drug that was encapsulated into the lipid matrix depended on the type of lipids used [32].

In the obtained AMD-SLNs, the lipid matrix was less ordered and had imperfections in its structure, leading to empty spaces available for the drug molecules to be encapsulated efficiently; thus, the total drug content in the lipid matrix was more than 80% (Table 2), which was due to the higher solubility of AMD in C888. The EE% of the SLNs’ formulations was found to be between 83.86 and 90.15% (Table 2). In applied experimental conditions, AMD loading in the lipid matrix resulted in an increase in SLN size, PDI values, and EE% values of 83.86–93.26%, proving the drug solubility in the lipid phase containing C888 and T as a surfactant, used as a penetration/permeation enhancer, and exemplifying their role in the development of lipid nanoparticles.

The higher values of EE% were observed for samples prepared with the same sonication amplitude but with a lower sonication cycle (formulations P1, P3, and P5 compared to P2, P4, and P6); therefore, the P1, P3, and P5 samples were considered for developing and optimizing. The drug loading of the AMD-SLNs ranged from 2.46 ± 0.015 to 5.28 ± 0.01%. In the obtained AMD-SLNs, C888 exhibited lower crystallinity and structural imperfections that created spaces for drug molecules, as expected. Furthermore, after cooling, C888 recrystallized in various polymorph forms (see DSC results).

### 3.4. Dynamic Light Scattering (DLS) for Polydispersity Index (PDI) and Zeta Potential (ZP) Results

**Figure 3:**
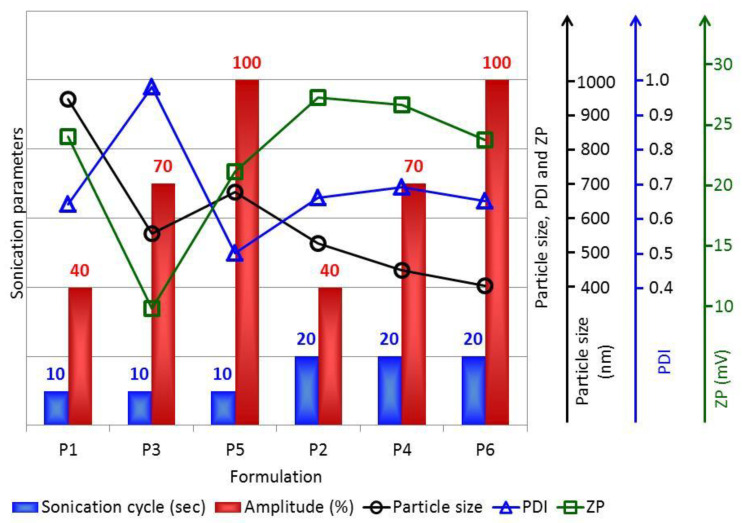
Comparative data obtained for the particle size, PDI, and ZP at different amplitudes and cycles of sonication of SLN encapsulating AMD.

The DLS method was used to determine the size of the AMD-SLNs, and the obtained results confirmed the nanoparticles’ formation. Moreover, this method provided information on the degree of dispersion and the ZP of the particles. The results presented in Table 3 and Figure 2 show that the size of the AMD-SLNs was in the range 405.06–675.00 nm, except for P1, which had AMD-SLNs of 944.44 nm, and the PDI values were below the threshold of 1. Moreover, from Figure 2, it is possible to observe bimodal distributions in almost all samples (except sample P3, where three modal distributions appear), one with low dimensions (almost all < 100 nm) and the other with dimensions as shown in Table 3. Nanoparticle systems with a ZP value in the range of –30 mv to –15 mV and in the range of +15 mV to +30 mV are considered stable colloidal suspension systems that prevent nanoparticle aggregation in accordance with SEM results. Suspended particles with PDIs greater than 0.1 imply large particle size distributions (0.498 ± 2.18 × 10^−2^–0.980 ± 2.01 × 10^−2^). Figure 3 compares the data obtained regarding particle size, PDI, and ZP.

Analyzing the data presented in Table 3 and Figure 3, it is observed that (i) for the same sonication cycle, as the sonication amplitude increased, the particle size decreased, with the exception of formulation P3, and (ii) for the same sonication amplitude, as the sonication cycle increased, the particle size also decreased, so the P5 and P6 samples had the smallest sizes in the particular conditions for obtaining each one. On the other hand, these two formulations proved to have the best morphological characteristics.

PDI is an indicator that measures the heterogeneity of the materials in aqueous solutions, with a smaller value indicating a narrower size distribution, which is most likely related to a smaller tendency of the AMD-SLNs to form aggregates [45].

For the same sonication cycle, the values of PDI increased for the sonication amplitude range of 40 to 70% and decreased for the sonication amplitude range of 70 to 100%. The lowest values were obtained for samples P5 and P6, which means a narrower distribution of particles for AMD-SLNs was obtained with a higher amplitude. At the same time, for the formulations obtained with a higher sonication cycle (P2, P4, and P6), the PDI values were much more uniform. Except for formulation P3, at the same sonication cycle and with an increase in amplitude, for formulations P5 and P6, there was a decrease in both the particle size (675.00 ± 59.12 nm–405.06 ± 37.43 nm, respectively) and the PDI (0.498 ± 2.18 × 10^−2^–0.650 ± 2.16 × 10^−2^, respectively), indicating that for these two formulations, the T amount was sufficient to emulsify the lipids with the aqueous phase, and to form AMD-SLNs, but also to stabilize and disperse them in the aqueous phase [46]. The studied particles were SLNs or nanosized lipid carriers, ranging from 405 to 944 nm, with the capability to encapsulate AMD molecules inside the lipid matrix.

The stability of the AMD-SLNs was also evaluated through ZP assessment. ZP values indicate the degree of repulsion between adjacent, similarly charged particles in dispersion, and the values could be correlated to the stability of colloidal dispersions. For molecules and particles that are small enough, a high ZP confers stability, i.e., the solution or dispersion will resist aggregation. When ZP is low, attraction exceeds repulsion and the dispersion breaks and flocculates [47]. Many experiments have demonstrated that not only electrostatic repulsion but also the steric stabilizer could impart stability to the AMD-SLNs’ dispersion [48]. For the studied AMD-SLNs, the ZP values were 9.68 ± 7.23 × 10^−1^ mV (for formulation P3) and in the range of 21.0 ± 7.06 × 10^−1^ to 27.1 ± 4.50 × 10^−1^ mV (for formulations P1, P2, P4, P5, and P6), indicating the stability of the AMD-SLNs due to steric stabilization. The DLS results were in good accordance with those obtained through SEM analysis.

Considering that the formulations obtained with a shorter sonication cycle show (i) higher EE% values and (ii) a narrower distribution of particles (except for the P3 formulation), we chose the formulations obtained with a shorter sonication cycle (P1 and P5) for further study, although the highest instability of all the studied AMD-SLN formulations, with a tendency to flocculate relatively quickly, was found in P3 (with a much lower ZP value, only 9.68 ± 7.23 × 10^−1^ mV, compared to the other AMD-SLNs, which had ZP values in the range of 21.0 ± 7.06 × 10^−1^–27.1 ± 4.50 × 10^−1^ mV, as shown in Table 3), though this formulation had the highest EE% value.

### 3.5. FT-IR Spectroscopy Results

The pure drugs, lipid excipients, surfactants, and AMD-SLN formulations (P1, P3, and P5) selected on the basis of their higher EE% and lower PDI values were studied to determine whether any interactions exist.

**Figure 4:**
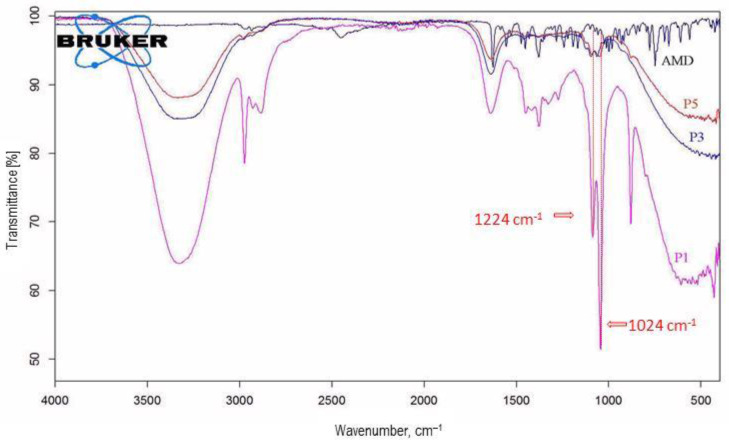
FTIR spectra of pure AMD and AMD-SLNs (P1, P3, P5).

The spectrum of pure AMD presented strong bands and had a great decrease in intensity in the AMD-SLN spectrum, as a consequence of the drug-loading process (P1, P3, and P5) (Figure 4). The AMD spectrum presented specific bands at 2960–2800 cm^−1^ for the –CH aliphatic group, 1631 cm^−1^ for the diaromatic C=O stretching, 1558 and 1529 cm^−1^ for the aromatic ring C=C quadrant stretching, 1477 and 1454 cm^−1^ for the aromatic ring C=C semicircle stretching, 1284 cm^−1^ for the ketonic C=O binding, 1245 and 1076 cm^−1^ for the aromatic ether C−O−C stretch, and 750 cm^−1^ for the aromatic C−H out-of-plane binding of the four adjacent aromatic hydrogen atoms. The AMD spectrum presented two bands related to tert-amine C−N at 1224 and 1024 cm^−1^. Those bands were also found in the spectrum of AMD-SLNs, as an indication of the encapsulation process.

**Figure 5:**
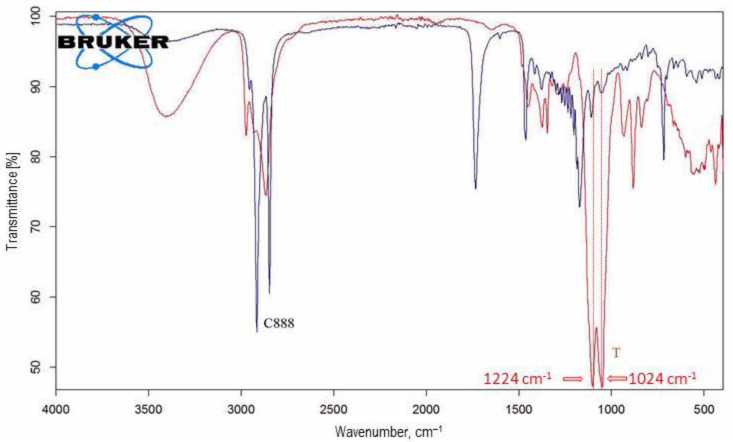
FTIR spectra of pure excipients (C888 and T).

For C888, T, and AMD-SLNs, the major bands for transmittance (%) were observed at 3392–3200 cm^−1^; these were assigned to the –OH groups stretching vibrations, while those at 2971 and 2871 cm^−1^ were assigned to the CH groups stretching vibrations. Other bands corresponding to the C-C groups stretching vibrations at 1380–724 cm^−1^ and to the C-O-C groups stretching vibrations at 1110–1070 cm^−1^ were observed in all excipients’ spectra (Figure 5).

In the AMD-SLNs’ spectra, similar bands could be observed at 2964–2934 cm^−1^ assigned to –CH aliphatic group stretching vibrations, 1630–1640 cm^−1^ assigned to diaromatic C=O stretching vibrations, and 1080–1050 cm^−1^ assigned to C−N from tertiary amines of AMD.

The band positions were characteristic to each type of AMD-SLN, which meant that there were some substituent interactions in those SLNs due to the appearance of host–guest interactions.

### 3.6. Near-Infrared (NIR) Spectroscopy Results

NIR spectroscopy is a non-destructive method widely used when analyzing materials. Small changes in the chemical structure and/or physical–chemical properties of materials cause spectral changes in diffusely reflected near-infrared radiation. In the case of NIR spectroscopy, the overtone and combination modes of the specific groups of materials are usually highly overlapped. As a consequence, the bands are very broad and the position, the width, and the height cannot be accurately estimated; therefore, the second derivative spectra are more appropriate for analysis.

**Figure 6:**
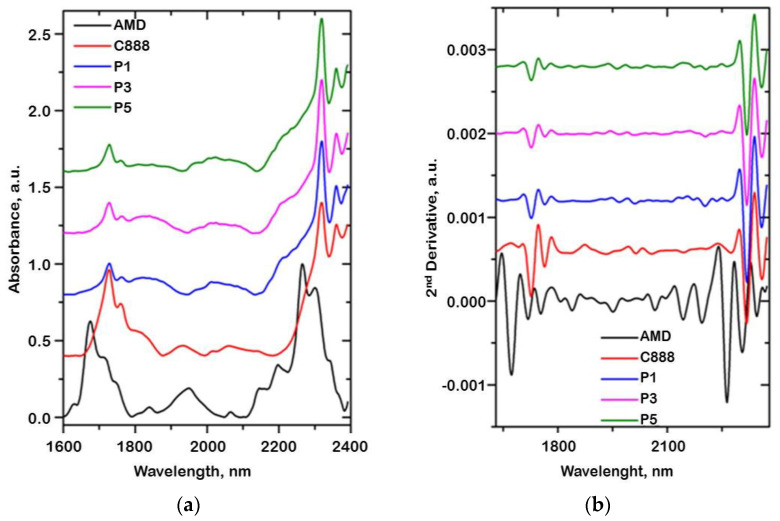
NIR spectra (**a**) and their second derivative (**b**) of AMD, C888, and AMD-SLNs (P1, P3, and P5).

In Figure 6a, the NIR spectra of the AMD, C888, and three AMD-SLNs of the drug-loading process (P1, P3, and P5) are presented. The spectra present the bands corresponding to the first overtones of C-H combination bands and second overtones of C-H and C_ar_-H stretching vibrations in the 1000–1480 nm and 1670–2000 nm spectral regions and the combination bands of C-H, C-O, and C=O stretching vibration of the acetyl groups in the 2000–2400 nm spectral region.

Due to the high overlapping bands, the spectral modifications in intensity of one band could be observed as a band shift or change in its width. Therefore, in order to clearly determine the component bands, the second derivatives of the NIR spectra were calculated (Figure 6b). From the second derivative spectra of the AMD-SNLs (P1, P3, and P5), the characteristic bands of AMD could be easily observed at 2032, 2066, 2095, 2142, and 2264 nm, characteristic to the stretching vibrations of CN, C=O, CH, and aromatic C-H and C-O groups. The characteristic bands of C888 at 1727, 1763, 2013, 2206, 2318, and 2360 nm corresponded to the stretching vibration of C=O groups, to the combination of CH and C=O groups and the second overtone of the bending vibration of the CH and CH_2_ groups, respectively. The presence of specific groups from AMD, C888, and T components in the P1, P3, and P5 formulations indicated either no interaction between them in the processing conditions or that they were very weak.

### 3.7. Thermogravimetry Results

**Figure 7:**
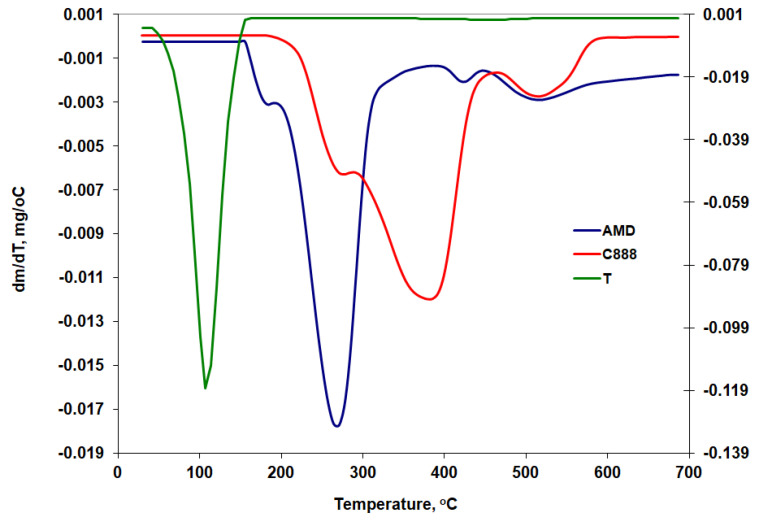
DTG curves for AMD, C888, and T components.

**Figure 8:**
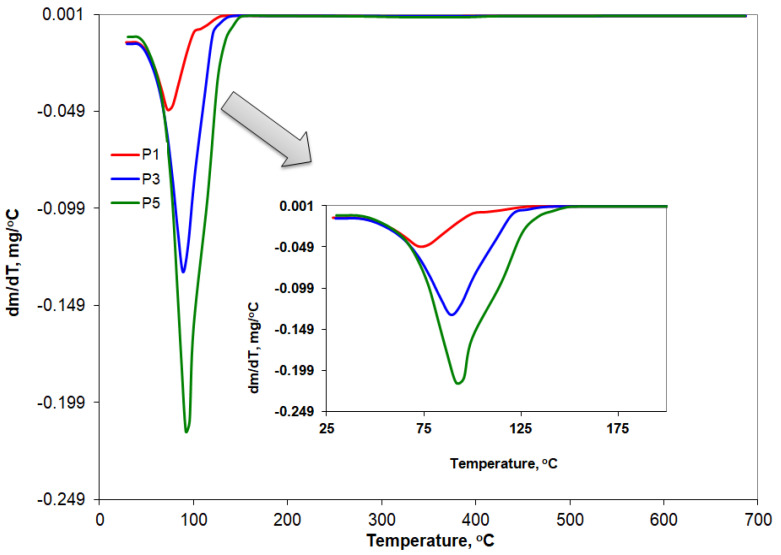
DTG curves for AMD-SLNs as P1, P3, and P5 samples.

The derivative thermogravimetric curves (DTG) are presented comparatively in Figure 7 for the pure substances (AMD, C888, and T) and in Figure 8 for the three AMD-SLNs (P1, P3, and P5) that resulted from the drug-loading process.

The main thermogravimetric characteristics are summarized below. The onset temperature of the mass loss processes (T_onset_), the temperature at which the processes reached their maximum rate (T_peak_), the temperature at which those processes ended (T_endset_), and the percentage of mass loss (Δw%) are shown in Table 4.

According to the data presented in Table 4, the thermal decomposition of AMD took place in four successive processes, with different percentages of mass loss starting at 167 °C. That value was slightly higher than that reported by other researchers (T_onset_ = 160 °C) who had studied the thermal decomposition of AMD under similar conditions, but with a different type of equipment [49]. Ledeti et al. [50] reported T_onset_ = 157 °C at the same heating rate but in the presence of air. The first process began just before the melting was completed, and it was consistent with data from a recent study published by Mhoumadi et al. [51], who applied the coupled TG-FTIR technique for the removal of water and 2-fluoroethanol. At higher temperatures, it continued with the elimination of triethylamine, hydrogen chloride, hydrogen iodide, etc. [52]. The most significant mass loss was found in the temperature range of 226–296 °C with a maximum at 268 °C. At 700 °C, a residue of 16.55% was collected at the end of the test. Yoshida et al. [53] reported a residual amount, 13%, at 750 °C for thermogravimetric curves recorded for AMD in nitrogen at a heating rate of 10 °C/min.

The degradation of glyceryl behenate (C888) took place in an inert atmosphere in three successive endothermic processes with peaks at 275, 383, and 512 °C. The amount of residue remaining at 700 °C was less than 1%. Passos et al. [52] reported a complete degradation of glyceryl behenate when the thermogravimetric curve was also recorded in a nitrogen atmosphere at a rate of 10 °C/min between 30 and 600 °C.

The analysis of the results indicates that up to 150 °C, the complete degradation of T took place through two successive processes, with an obvious peak at 108 °C. In the case of the P1, P3, and P5 AMD-SLNs, according to the DTG curves in Figure 8 and the data presented in Table 4, there was a shift of the peak from 73 °C to 93 °C and also an increase in the temperature from 114 °C to 134 °C, at which the mass loss was complete. It was found that, in the case of the three formulations, there was a quantity of residue between 2.49 and 5.14% at the end of the test, which could be associated with the presence of AMD.

### 3.8. Differential Scanning Calorimetry (DSC) Results

**Figure 9:**
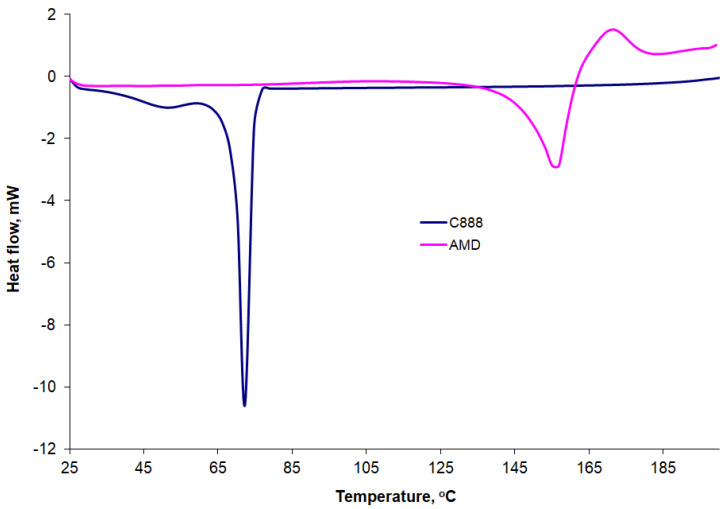
DSC curves for AMD and C888 pure substances at a 10 °C/min heating rate.

The DSC curves recorded for the AMD and C888 pure substances at a heating rate of 10 °C/min are presented comparatively in Figure 9. We recorded a melting peak for AMD at 156 °C and an exothermic peak at 171 °C, which indicated the onset of thermal decomposition. Those results were in agreement with reports from the literature [49,52,54,55]. The peaks and enthalpy variations recorded at a heating rate of 10 °C/min are shown comparatively in Table 5. The melting enthalpy obtained was 70.57 J/g, while Ledeti et al. [50] reported a melting enthalpy of 65.4 J/g for AMD recorded under the same conditions, namely a nitrogen atmosphere and heating rate of 10 °C/min. The DSC curves recorded for C888 showed two endothermic peaks. The most prominent peak was at 72 °C, with a melting enthalpy of 120.52 J/g. Chaiya et al. [55] reported a melting peak of 75.3 °C for C888 and an enthalpy of 117.4 J/g for DSC curves recorded under the same conditions.

**Figure 10:**
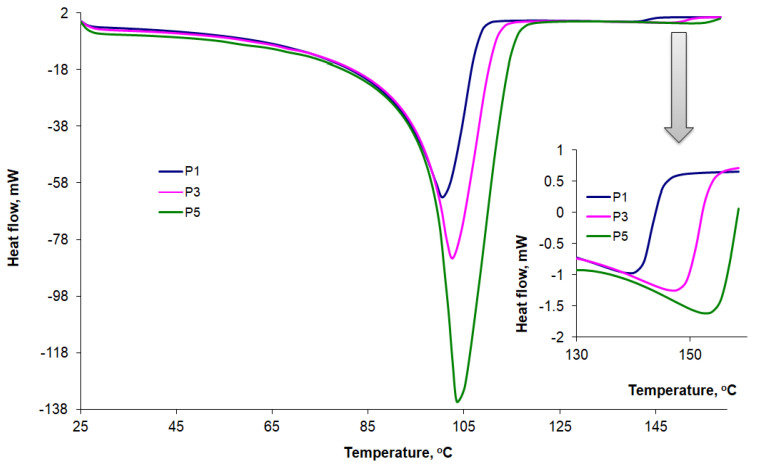
DSC curves for AMD-SLNs (P1, P3, and P5) at a 10 °C/min heating rate.

The DSC curves recorded at a heating rate of 10 °C/min for P1, P2, and P3 AMD-SLNs are shown comparatively in Figure 10, and the characteristic parameters are presented in Table 5. Wide peaks were identified at around 100 °C, along with a series of peaks at temperatures ranging from 141 °C to 154 °C that could be associated with the presence of AMD in formulations. Some of our DSC results are in accordance with those found in the literature. The authors of [56] investigated the stability of different lipid excipients, including Compritol 888, in SLNs and found that the lattice arrangement of Compritol 888 ATO crystals generally comprised small amounts of the unstable polymorphic form that disappeared after thermal stress.

**Figure 11:**
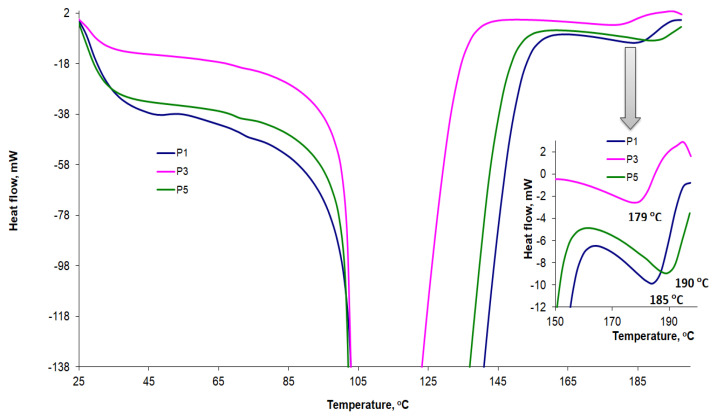
DSC curves for AMD-SLNs (P1, P3, and P5) at a 50 °C/min heating rate.

As highlighted in this study and also reported in the literature [49,51,52,55,57], if the heating rates in the DSC analysis were not high enough, AMD began to degrade before the melting process was completed. Mhoumadi et al. [51] recently proposed to remove that shortcoming by increasing the heating rate. When the heating rate was increased from 10 to 60 °C/min, the degradation occurred immediately after the melting process. Figure 11 shows the results obtained when we increased the heating rate for AMD-SLNs to 50 °C/min. The peaks indicating the presence of AMD shifted to higher temperatures between 179 °C and 190 °C, and the degradation processes occurred at higher temperatures, after the melting process was complete.

### 3.9. In Vitro AMD Release from the AMD-SLNs

**Figure 12:**
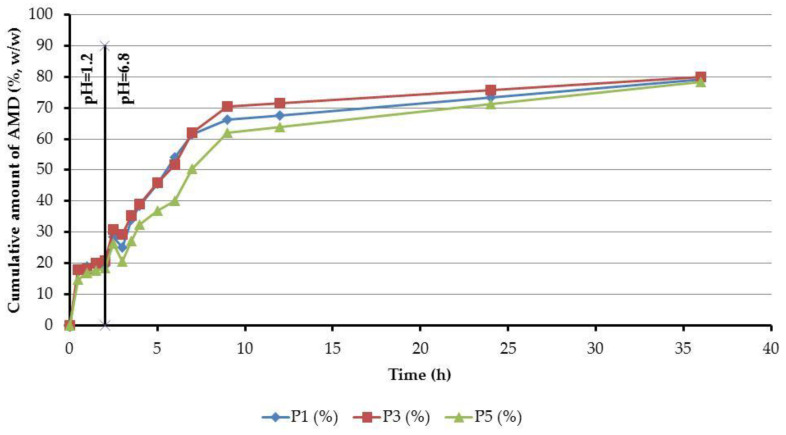
Release profiles of AMD from AMD-SLNs (P1, P3, and P5).

An in vitro dissolution test was performed in simulated gastrointestinal fluids, and it showed no significant differences between the AMD-SLNs (Figure 12).

Two-step release profiles were recorded for all samples. The first one indicated a faster release; then, in the second step, a slow release took place, reaching a maximum value. In the case of the dissolution test performed in simulated gastric fluid with pH = 1.2, there was an AMD release of 19.99% (P1), 17.99% (P3), and 14.75% (P5) at the first sampling at 30 min. After 2 h, in the simulated gastric fluid with pH = 1.2, we observed an AMD release of 19.24%, 20.79%, and 18.41% for P1, P3, and P5, respectively. The AMD release of approximately 50% in simulated intestinal fluid with pH = 6.8 was achieved after 5.5 h (P1), 5.7 h (P3), and 6.9 h (P5). After 36 h, the AMD release was 79.14% (P1), 80.07% (P3), and 78.54% (P5).

The analyzed samples fall into the release profile for extended-release dosage forms, according to “2.9.3. Dissolution test for solid dosage forms” and “5.17. Recommendations on methods for dosage forms testing” from the European Pharmacopoeia, 10th edition [41]. This fact is, thus, an argument in favor of obtaining pharmaceutical forms with controlled release.

### 3.10. Kinetic Release of AMD from AMD-SLNs

The kinetic release parameters were calculated using the Korsmeyer–Peppas model [44], and they are reported in Table 6. The table refers to the initial (0–120 min) and second (120–360 min) stages of drug release.

The obtained value of *n_r_* = 0.09 in the first kinetic release stage for P1 was characteristic of a pseudo-Fickian drug transport mechanism, while, in the second stage, the calculated value of *n_r_* = 1.008 fit the transport of a case II mechanism. There was a shift in the drug transport mechanism from Fickian to non-Fickian behavior for P3 and P5 between the first and the second kinetic release stages, when the values of the release exponent were below 0.5 (*n_r_* = 0.115 and *n_r_* = 0.156) in the first kinetic release stage and ranged between 0.5 and 1 (*n_r_* = 0.711 and *n_r_* = 0.700) in the second kinetic release stage.

The kinetic constant *k_r_* values, listed in Table 6, were significantly higher in the first kinetic release stage than in the second one, suggesting that the AMD diffusion degree from the AMD-SLNs was much higher in the initial stage of release in the SMGF with pH = 1.2. That behavior was observed for all three AMD-SLNs, namely P1, P3, and P5.

## 4. Conclusions

AMD-loaded SLNs (AMD-SLNs) were produced through a hot homogenization emulsification–ultrasonication technique and a selected composition including C888 as the lipid matrix and T as the surfactant, and the solvent for penetration enhancement was prepared. The ratio between AMD, the lipid excipient C888, and T was maintained constant, but various ultrasonication conditions were applied. The AMD-SLNs were tested for controlled drug release and the drug-loading capacity of the trapped active substance. The formulations obtained at a 100% sonication amplitude showed an entrapment efficiency (EE%) of 85% *w*/*w* and drug loading of 2.50–5.20%, *w*/*w*. The obtained AMD-SLNs were spherical, smooth, uniform particles with no imperfections and no fissures. According to the FT-IR and NIR analyses, in the encapsulated AMD samples, weak interactions between the active pharmaceutical substance and the two excipients, C888 and T, took place. The peaks recorded in the DSC analysis indicating the presence of AMD shifted to higher temperatures, between 179 °C and 190 °C, and it was found that the degradation processes occurred at higher temperatures, after the melting process was complete.

Small PDI values (except P3) indicated a narrower size distribution, which is most likely related to a small tendency of the AMD-SLNs to form aggregates. The in vitro dissolution test produced good results for the AMD-SLNs, and they proved to be suitable for the prolonged and controlled release of AMD. After 2 h in simulated gastric fluid with pH = 1.2, the AMD release was 18%; it was approximately 50% (*w*/*w*) in the simulated intestinal fluid with pH = 6.8 after 7 h and 80% after 36 h. The in vitro kinetic study revealed a complex mechanism of release occurring in two steps through a pseudo-Fickian mechanism. Taking into account these data, it can be concluded that the obtained AMD-SLNs (except P3) showed optimal pharmaceutical–technical properties and had the greatest potential to be used in controlled-delivery oral pharmaceutical products with amiodarone. This study represents an initial step for future studies that could more extensively explore the dose–response relationship of AMD-loaded SLNs on lipid profile modulation for the assessment of their biocompatibility and potential therapeutic applications.

The P5 formulation showed optimal pharmaceutical–technological properties, and it had the greatest potential to be used in oral pharmaceutical products for the controlled delivery of amiodarone.

## Figures and Tables

**Table 1:** Preparation of AMD-SLNs.

Formulation of AMD-SLNs	AMD (%, *w*/*w*)	Compritol 888 (%, *w*/*w*)	Transcutol (%, *w*/*w*)	Water (%, *w*/*w*)	Amplitude (%)	Sonication Cycle (sec)
P1	0.13	5	2	92.87	40	0.5
P2	0.13	5	2	92.87	40	1.0
P3	0.13	5	2	92.87	70	0.5
P4	0.13	5	2	92.87	70	1.0
P5	0.13	5	2	92.87	100	0.5
P6	0.13	5	2	92.87	100	1.0

**Table 2:** DL% and EE% of AMD-SLNs (average and standard deviation, n = 3).

AMD-SLN	Determined Concentration (μg/mL)	DL%	EE%
P1	1172	5.16 ± 0.02	90.15 ± 2.23
P2	1112	5.28 ± 0.01	85.61 ± 2.38
P3	1213	2.46 ± 0.015	93.26 ± 3.12
P4	1085	5.21 ± 0.02	83.86 ± 2.94
P5	1150	2.51 ± 0.01	88.46 ± 1.55
P6	1114	5.15 ± 0.02	85.65 ± 1.65

**Table 3:** Physical characteristics of AMD-SLNs (mean ± SD, n = 3).

Formulations AMD-SLNs	Amplitude (%)	Sonication Cycle (sec)	Size (nm)	PDI	ZP (mV)
P_1_	40	0.5	944.44 ± 82.33	0.641 ± 3.43 × 10^−2^	23.9 ± 5.51 × 10^−1^
P_2_	40	1	527.78 ± 48.86	0.660 ± 3.23 × 10^−2^	27.1 ± 4.50 × 10^−1^
P_3_	70	0.5	555.56 ± 48.34	0.980 ± 2.01 × 10^−2^	9.68 ± 7.23 × 10^−1^
P_4_	70	1	450.00 ± 42.52	0.690 ± 3.17 × 10^−2^	26.5 ± 5.42 × 10^−1^
P_5_	100	0.5	675.00 ± 59.12	0.498 ± 2.18 × 10^−2^	21.0 ± 7.06 × 10^−1^
P_6_	100	1	405.06 ± 37.43	0.650 ± 2.16 × 10^−2^	23.6 ± 5.02 × 10^−1^

**Table 4:** Main thermogravimetric characteristics for AMD, C888, T, and AMD-SLNs (P1, P3, and P5).

Sample	Stage	T_onset_ (°C)	T_peak_ (°C)	T_endset_ (°C)	Δw (%)	Residue
AMD	I	167	180	226	8.03	16.55
	II	226	268	296	48.23	
	III	412	423	436	6.09	
	IV	472	511	593	21.10	
C888	I	239	275	325	29.14	0.82
	II	325	383	417	57.18	
	III	484	512	570	12.86	
T	I	69	108	120	84.85	0
	II	120	-	140	15.15	
P1	I	49	74	83	86.15	5.14
	II	83	106	114	8.71	
P3	I	53	89	99	94.11	2.49
	II	99	-	123	3.40	
P5	I	56	93	104	92.69	2.52
	II	104	-	134	4.79	

**Table 5:** Peaks and enthalpies recorded at 10 °C/min heating rate.

Sample	T_peak_ (°C)		ΔH (J/g)	
	1	2	1	2
AMD	156	171	−70.57	+20.70
C888	51	72	−8.29	−120.52
P1	101	141	−1802.48	−11.14
P2	102	149	−1676.56	−9.75
P3	104	154	−1706.40	−9.83

**Table 6:** AMD release kinetic parameters for the three samples.

AMD-SLNs	First Stage of Kinetic Release Profile (0–120 min)				Second Stage of Kinetic Release Profile (120–360 min)			
	n_r(1)_	$R_{n_{1}}^{2}$	k_r(1)_ (min^−n^)	$R_{k_{1}}^{2}$	n_r(2)_	$R_{n_{2}}^{2}$	k_r(2)_ (min^−n^)	$R_{k_{2}}^{2}$
P1	0.0905	0.8253	0.1266	0.9980	1.0081	0.9116	0.00145	0.9840
P3	0.11528	0.7787	0.11876	0.9961	0.71115	0.9987	0.0079	0.9998
P5	0.15667	0.9817	0.08714	0.9994	0.70063	0.9210	0.00664	0.9938

n_r_ = release exponent; k_r_ = release rate constant; $R_{k}^{2}$ and $R_{n}^{2}$ = correlation coefficients corresponding to the slope obtained for determination of n_r_ and k_r_.

## Data Availability

Not applicable.
